# Editorial: “Investigations into the use of organic and inorganic compounds as chemotherapeutic agents’’

**DOI:** 10.3389/fmolb.2023.1326680

**Published:** 2023-11-23

**Authors:** Paras Nath Yadav, Ana Isabel Matesanz

**Affiliations:** ^1^ Central Department of Chemistry, Tribhuvan University, Kathmandu, Nepal; ^2^ Inorganic Chemistry Department, Autonomous University of Madrid, Madrid, Spain

**Keywords:** metallodrug, biomolecules, cancer, chemotherapy, inorganic, organic

Cancer is one of the major causes of death the worldwide, especially in developing countries (Siegel et al., 2020). Numerous anticancer drugs available have a high price which people from the poor section of society can hardly afford (Prasad et al., 2017). Besides, some of the anticancer drugs have harmful side effects, resulting in poor resistance from the body (Basak et al., 2022). In recent years, numerous ongoing research works, including Cancer Genetics and Genomics, Cancer Immunotherapy and Vaccines, Cancer Metabolism, Cell Signaling and Regulation, Cell-Based Therapy, Computational Oncology, Immunology, Metastasis and Drug Resistance, Solid Tumor Oncogenesis, and Targeted Therapy have generated new hope among cancer patients. All of these cancer related research projects highlight the need of continuing these research works. In addition, universities and research institutes need to promote research works on cancer related drug, origin of cancer, mechanism of drug action, cancer control, public awareness, among others. Findings of these research works should be publicized through scholarly journals. In this line of vision, our journal provides the oncology scientists with a platform for publication of their quality research works that substantiate people’s understanding of therapeutic strategies in cancer. Strict quality control procedures are adopted before acceptance of articles, to maintain the quality of papers and to ensure the integrity of data. The journal covers a range of cancer related Research Topic to target a broad audience of cancer researchers encouraging submissions of research works from across the world.

This volume of our journal covers key areas of cancer research such is the use of organic and inorganic compounds as potential chemotherapeutic agents. Developing new drugs involves synthesizing novel compounds, identifying new drug targets, and optimizing their pharmacological properties. In general, metallodrugs possess versatile electronic and structural properties and understanding the nature of their interactions with biomacromolecules is of utmost importance (Anthony et al., 2020). Articles in this volume include; coordination chemistry of Ru(II) and Ni(II) and their anticancer activity, DNA interaction as well as molecular docking. Activity of the Ru(II) complexes is related to lipohilicity, cellular localization, and specific interactions with biomolecules. The complexes localize in the nuclei of cancer cells and co-locate with DAPI on DNA (Nyong-Bassey et al.). Nickel (II) complexes displayed *in vitro* promising anticancer activity. Apart from this, molecular docking simulations were studied to evaluate the properties and interactions of the free dithiocarbazates ligands as well as their Ni(II) complexes with selected proteins and DNA (Cavalcante et al.). Another series of research focus on organic molecules like iron chelator SK4 which has been found cytotoxic in tumor cell lines. This molecule represents the first iron chelator that gains cell entry through the large neutral amino acid transporter (LAT1). Furthermore, the authors demonstrated that this new agent targets both iron and amino acid metabolism dysregulation (Abdelaal et al.). Some of the authors have explored natural compounds, including ursolic acid, digoxin and casticin as potential anticancer agents (Zou et al.; Carbone et al.). Ursolic acid and digoxin have been at clinical trials for treatment of different cancers, such as prostate cancer, pancreatic cancer and breast cancer. Previous studies have demonstrated that ursolic acid and digoxin are potential RORγt antagonists in modulating the functions of immune cells such as Th17 cells. The findings suggest that RORγ is a direct target of ursolic acid in cancer cells that helps select patients with tumors that likely respond to ursolic acid treatment. One notable paper established the role of the copper transporter CTR1 in the accumulation of the widely used anticancer metallodrug, cisplatin (Schoeberl et al.). They showcased the competitive impact of cisplatin treatment on copper homeostasis at the single-cell level by LA-ICP-TOFMS. Meta-analysis work on combined chemotherapy with anti-EGFR antibody as an effective therapy has also been reported on patient where surgical operation was not favorable. The meta-analysis aimed to assess the clinical efficacy of chemotherapeutic triplet-drug regimen combined with anti-EGFR antibody in patients with initially unresectable metastatic colorectal cancer (mCRC).

The journal includes a relevant review report on obese patients with malignant tumor. Certain tumors are associated with obesity, the clinical management of obese patients is often complex. The paper has analyzed the dosage of anti-tumor drugs in obese patients, in an anticipation of providing more data for the clinical treatment of obese patients with malignant tumors. It contains various research findings useful for researchers and academicians working with cancer related drug development.

According to our Journal policy, the manuscripts have been peer-reviewed and revised to address the comments from reviewers before they are finally accepted. We could not include all the manuscripts in view of the number of articles to be included in this journal. Those authors whose articles have been rejected for this time can submit their manuscripts for the next volume of this journal. We are very hopeful to find manuscripts that provide original and innovative ideas that can contribute to the advancement of science and innovation in medical technology in the greater purpose of humanity.

I would like to express my deep gratitude to reviewers and Editors for their contribution to reviewing the manuscript meticulously. I would like to appreciate the Managing Editors and administrative staff for their significant contribution to bring forth this publication.

We hope for receiving high quality manuscripts from the authors for the next volume.
